# Are physical and mechanical properties of 3D resins dependent on the manufacturing method?

**DOI:** 10.1007/s10266-024-00985-3

**Published:** 2024-08-13

**Authors:** Fabio Rizzante, Hannah Hales, Sorin Teich, Adilson Yoshio Furuse, Gustavo Mendonça, Christian Brennes

**Affiliations:** 1 Assistant Dean for Innovation, James B. Edwards College of Dental Medicine, Medical University of South Carolina, Charleston, SC 29425 USA; 2 Dentist, Private Practice, Charleston, SC USA; 3 Associate Dean for Clinical Affairs, James B. Edwards College of Dental Medicine, Medical University of South Carolina, Charleston, SC USA; 4 Associate Professor, Department of Operative Dentistry, Endodontics and Dental Materials, Bauru School of Dentistry, University of São Paulo, São Paulo, SP Brazil; 5 Professor, Department of General Dentistry, School of Dentistry, Virginia Commonwealth University, Richmond, VI USA; 6 Associate Professor, Department of Advanced Specialty Sciences, James B. Edwards College of Dental Medicine, Medical University of South Carolina, Charleston, SC USA

**Keywords:** 3D printing, Resins, Digital dentistry

## Abstract

This research analyzed the effect of the manufacturing method on the flexural strength and color stability of 3D-printed resins used for producing indirect restorations. For this, two dental restorative biocompatible resin materials, OnX (OnX, SprintRay) and CB (Crown and Bridge, Dentca), were divided into 2 groups according with manufacturing method (printed with a Pro95 3D printer – SprintRay; and not printed, with samples obtained with the fluid resin being poured on PVS molds for further light activation in the post-curing process), and subdivided into 2 groups according to the post-curing method: VG (Valo Grand, Ultradent Products) for 120 s and PC (Procure 2, SprintRay). Bar-shaped samples were used to evaluate the flexural strength 24 h after storage in distilled water at 37 °C using a universal testing machine. Disk-shaped samples were used to evaluate the color stability with a spectrophotometer at baseline, after 1–7 days in dark dry storage at 37 °C, and after 1 day of artificial aging in water at 60 °C. Data were evaluated using 3-way ANOVA (flexural strength) and 4-way repeated measures ANOVA (color stability), followed by the Tukey’s HSD test (*α* = .05). Flexural strength showed significant results for resin (*p* < .001), while manufacturing and post-curing methods were not significant (*p* > .05). The interaction effects between resin and manufacturing method (*p* = .978), and between resin, manufacturing method and post-curing method (*p* = .659) were not significant. In general, OnX showed higher flexural strength values than CB, regardless of manufacturing method or post-curing protocol. Color stability results showed significant results for resin (*p* < .001), time (*p* < .001), resin and time (*p* = .029), and resin and curing method (*p* < .001), but no differences considering resin and manufacturing mode (*p* = .87), or resin, manufacturing method and curing method (*p* = .35). In general, OnX showed a higher color change than CB, longer storage times resulted in increased color change for both materials, and CB cured with VG showed lower color alteration than CB cured with PC2. The manufacturing method (3D printed or not 3D printed) does not seem to influence the flexural strength and color stability of 3D printed resins. This may indicate that, at least from a physical–mechanical perspective, the final properties of the material are mainly dependent on the post-curing process.

## Introduction

Additive manufacturing or 3D printing is becoming increasingly popular in the daily clinical practice due to its versatility, lower cost, and adequate accuracy when compared with traditional subtractive manufacturing (or milling) [1–8]. The use of 3D printers allows the conversion from a digital treatment plan to an analogic/real life outcome, resulting in a full integration with the digital workflow [2–13].

Most 3D printers used in dentistry are based on Stereolithography (SLA) or Digital Light Processing (DLP). While in SLA, a laser beam cures each individual layer, in DLP, a digital projector screen is used to project the entire layer, resulting in faster printing times. After cured, samples are washed, usually in 99% isopropyl alcohol, followed by post-curing in various combinations of curing unit, time, temperature, immersion in media, among others [6, 7, 9].

Resins currently available on the market for preparing 3D-printed dental restorations are considered biologically safe [13]. Nevertheless, this safety depends on the 3D printing workflow as resins for 3D printing contain more photo-initiators when compared with conventional resin composites [1, 14, 15]. This results in the presence of unreacted photo-initiators and monomers after the printing process, which may be minimized during the post-polymerization processes [1, 14, 15].

With the rapid increase in the number of available 3D printers and post-polymerization devices, along with a lack of standardization about printing workflows, there is increasing concern about the influence of the different components of the 3D printing workflow in the properties of 3D printed parts [1–5, 7–15]. This concern becomes even more critical considering that there are several 3D printers available in the market, ranging from very affordable, open-platform, hobby-like 3D printers, to professional grade 3D printers with closed-platform and completely validated workflows [13–15].

Literature reports both printing parameters and post-processing methods influence the physical–mechanical properties of 3D printed parts [2, 3, 6, 9, 13, 14, 16–23]. Nevertheless, most studies used open-platform 3D printers, different curing chambers with different curing times, in a not fully validated workflow. This lack of standardization prevents the understanding of how, or if, the 3D printer parameters and the post-polymerization parameters could influence the final properties of a 3D printed part. Therefore, it is unclear whether different 3D printers could influence the physical–mechanical properties of 3D printed parts if an adequate post-curing protocol were to be used.

In order to identify if the 3D printer would impact the physical–mechanical properties of 3D printed resins, this study evaluated the flexural strength and color stability of 2 resins for 3D printing, considering 2 situations: a fully validated 3D printer + different post-curing methods compared to no previous 3D printing + different curing methods. The null hypothesis tested was that there would be no difference between 3D printed and non-3D printed parts after the post-polymerization processes.

## Materials and methods

This study evaluated three factors: resins (in two levels), manufacturing methods (in two levels, i.e., printed and not printed), and post-curing methods (in two levels). The materials used in this study are listed in Table 1. This study had as response variables the 3-point flexural strength and the color stability.

**Table 1:** Materials used in this study

Material	Manufacturer	Description
OnX bleach	SprintRay	3D printed resin for final fixed and removable restorations
Crown and Bridge BL1	Dentca	3D printed resin for temporary crowns and bridges
Pro 95 3D printer	SprintRay	DLP 3D printer. Used with the respective resin profiles
Procure 2	SprintRay	42 LEDs-385 nm, up to 80 °C. Used with the respective resin profiles
Valo grand	Ultradent	120 s LED 385–515 nm Standard mode–1000mW/cm^2^

### Samples preparation

Two dental biocompatible 3D printing restorative resin materials, OnX (OnX, SprintRay) and CB (Crown and Bridge, Dentca), were divided into 2 groups according with manufacturing method (printed with a Pro95 3D printer – SprintRay, and not printed, with samples obtained with the fluid resin being poured on PVS molds for further light-activation in the post-curing process), and subdivided into 2 groups according to the post-curing method: VG (Valo Grand, Ultradent Products) for 120 s and PC (Procure 2, SprintRay) following the respective resin profiles recommended by the manufacturer. A total of 160 samples were obtained, 80 for each test (*n* = 10 for each subgroup and test).

#### Printed samples

For the samples printed using the Pro 95 3D printer, a 3D bar (2 × 2x10 mm) and a 3D disk (8 × 2 mm) were created using an open-source software (Meshmixer), exported as stereolithography (.stl), and imported to dashboard software (SprintRay) [6]. All samples were printed at 90°, with 100um layer thickness [6], with supports set up to medium and localized at the sides of the samples, avoiding areas of interest for the subsequent tests. The respective resin profiles, automatically provided by the software, were used for printing. After printed, OnX samples were sprayed with 99.5% isopropyl alcohol (IPA) and air blasted to removed uncured resin following manufacturer instructions, while CB samples were washed using the standard cycle of Pro Wash 2 (SprintRay). After washed, samples were post-cured following the proposed post-curing methods (VG and PC).

#### Non-printed samples

**Fig. 1:**
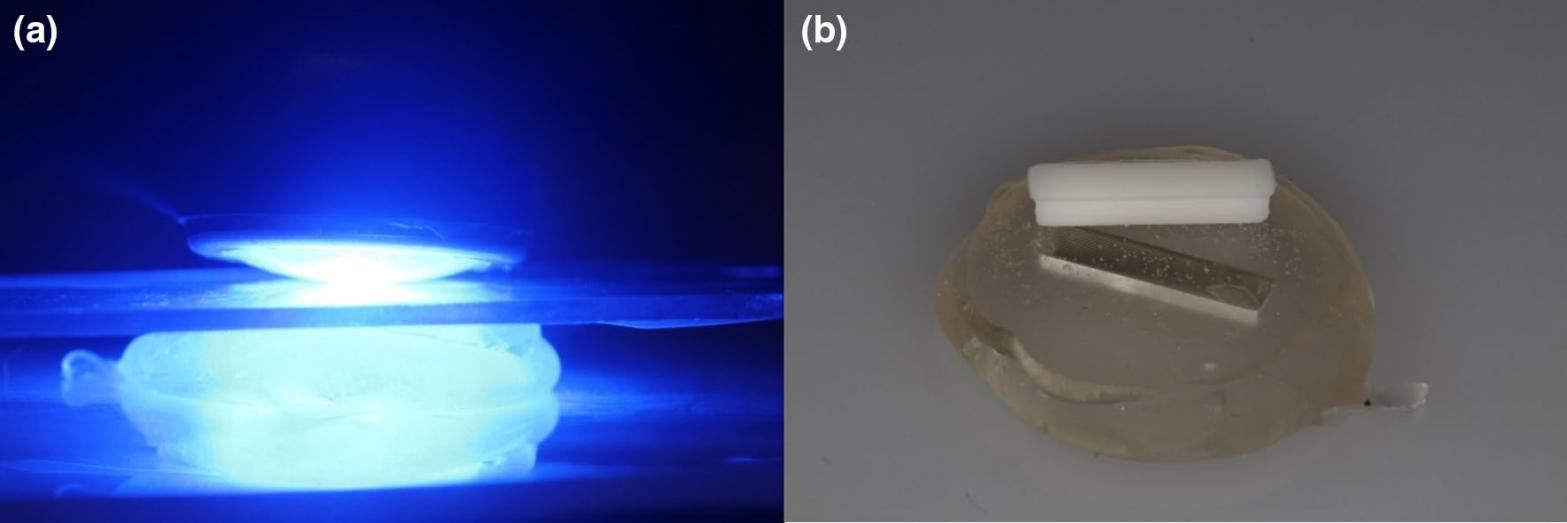
Manufacturing of non-printed bar samples. Light-curing with Valo Grand for 120 s (**A**), polymerized sample before removal of excess material (**B**)

For non-printed samples, a mold was created using a transparent PVS material (Memosil 2, Kulzer) based on the 3D printed disks and bars. The respective resins for 3D printing were inserted into these molds using a disposable syringe, a mylar strip was used to standardize the surface and sample dimension, and the samples were cured following the proposed curing methods (Figs. 1A and B). Excesses of material were removed using a sequence of 400-, 800- and 1200-grit sandpaper. Each sample was measured using a digital caliper to confirm the dimensions were accurate.

### Tests

#### Flexural strength test

Flexural strength was assessed 24 h after storage in distilled water at 37 °C using a universal testing machine. For this, a 3-point bending test device, with an 8 mm span, was adapted to a universal testing machine, and samples were tested in a 1 mm/min downward movement until fracture. The force in Newtons (*N*) was recorded and converted to MPa following the formula:

$${\text{FS}}\, = \,{\text{3Fl}}/{\text{2bd}}^{{2}} ,$$

where FS is the flexural strength in MPa; F the loading force at the fracture point in *N*, l the length of the support span (8 mm), and b and d the width and thickness of the specimen cross-section, measured with a digital caliper, respectively.

#### Color stability test

The color stability was evaluated using a spectrophotometer (Vita Easyshade V, Vita Zahnfabrik) at baseline, after 1–7 days in dark, dry storage at 37 °C, and after 1 day of artificial aging in deionized water at 60 °C [6, 7]. For each time point, the spectrophotometer was calibrated using the built-in calibration tool. Samples were positioned over a white background, dried with absorbent paper when needed, and evaluated with the tip of the device positioned perpendicular to the sample surface. Two measurements per specimen were taken until both results agreed. Degree of color change was calculated from:

$$\Delta {\text{E}}\, = \,\left[ {\left( {\Delta {\text{L}}*} \right)^{{2}} \, + \,\left( {\Delta {\text{a}}*} \right)^{{2}} \, + \,\left( {\Delta {\text{b}}*} \right)^{{2}} } \right]^{\raise.5ex\hbox{$\scriptstyle 1$}\kern-.1em/ \kern-.15em\lower.25ex\hbox{$\scriptstyle 2$} } ,$$

where ΔL*, Δa*, and Δb* represent the differences between the readings of the color parameters between the baseline and the different time points.

### Statistical analysis

The data were tabulated and evaluated for normality using Kolmogorov–Smirnov test, followed by 3-way ANOVA for flexural strength (resin, manufacturing method and post-curing method) and 4-way repeated measurements ANOVA for color stability (resin, manufacturing method, curing method, and time), followed by the Tukey HSD test. All statistical analyses were conducted adopting a global level of significance of 5%.

## Results

**Fig. 2:**
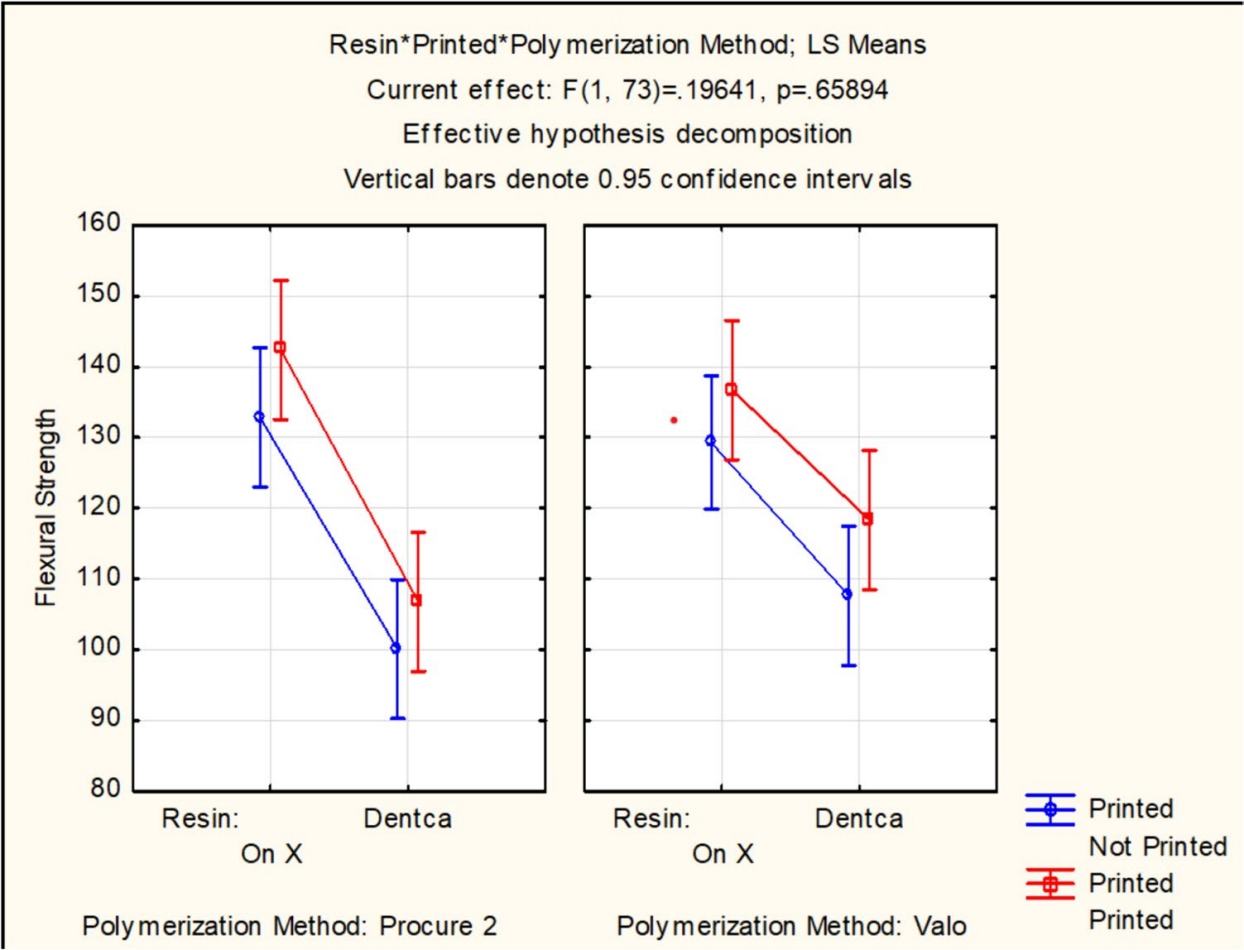
Flexural strength, in MPa, considering the factor resin, manufacturing method and curing method

Flexural strength (Table 2) showed significant results for resin (*p* < 0.001). The interaction effects between resin and manufacturing method (*p* = 0.978), and between resin, manufacturing method and post-curing method (*p* = 0.659) were not significant. In general, OnX showed higher flexural strength values than CB, regardless of manufacturing method or post-curing protocol (Fig. 2).

**Table 2:** Flexural Strength (std dev), in MPa, for tested groups

Resin	Flexural strength MPa
OnX PC2	137.24 (29.75)AB
OnX VG	130.43 (15.49)AB
OnX printed PC2	142.4 (17.99)A
OnX printed VG	136.71 (11.58)AB
CB PC2	88.82 (19.69)C
CB VG	99.93 (16.6)C
DP printed PC2	106.79 (7.42)C
CB printed VG	118.34 (15.33)BC

Different letters mean statistically significant differences between groups. Lowercase letters mean difference intra group (between columns)

Different uppercase letters mean statistically significant differences (*p* < .05)

**Fig. 3:**
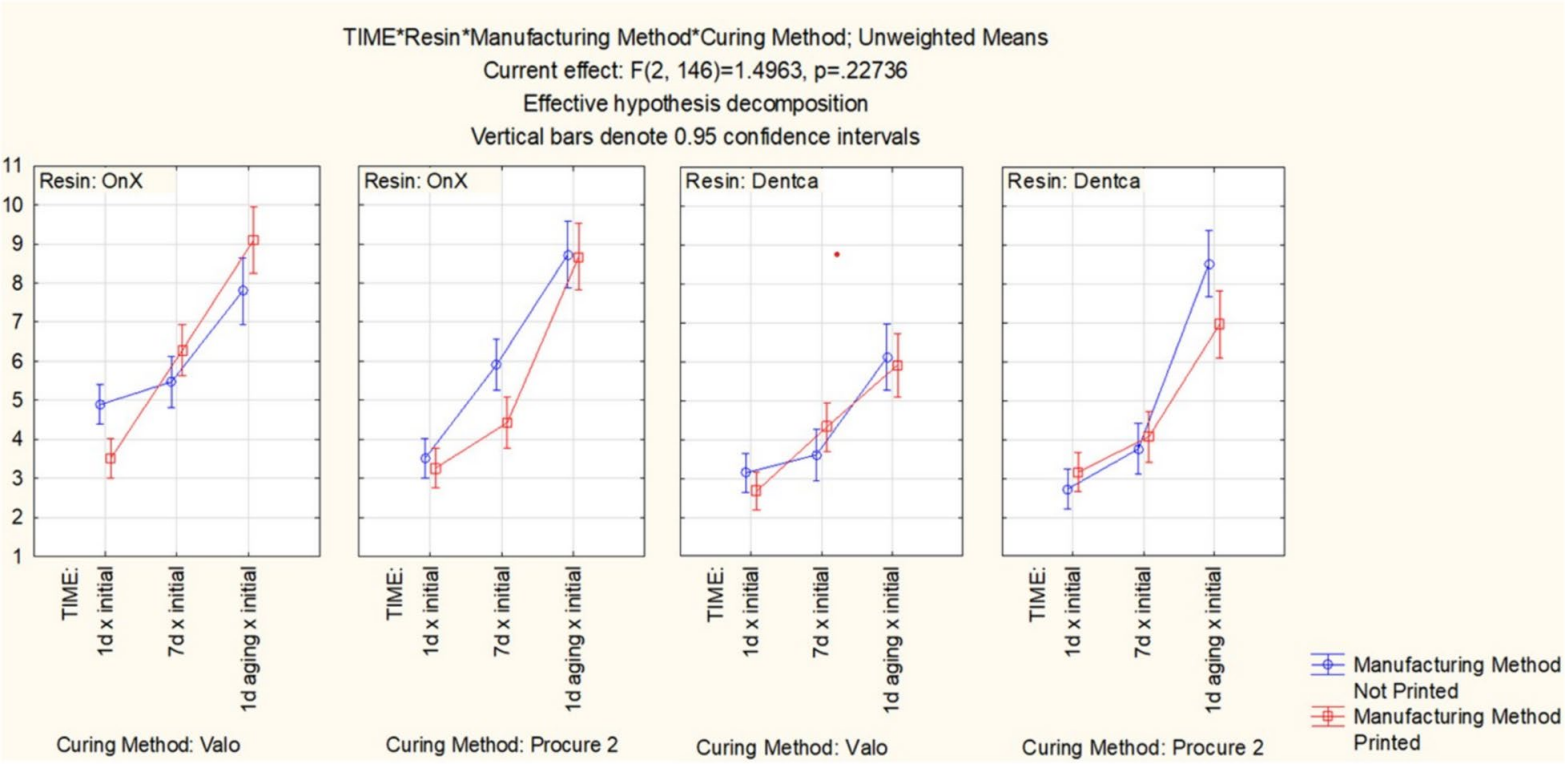
Color stability considering the different groups and time points

**Fig. 4:**
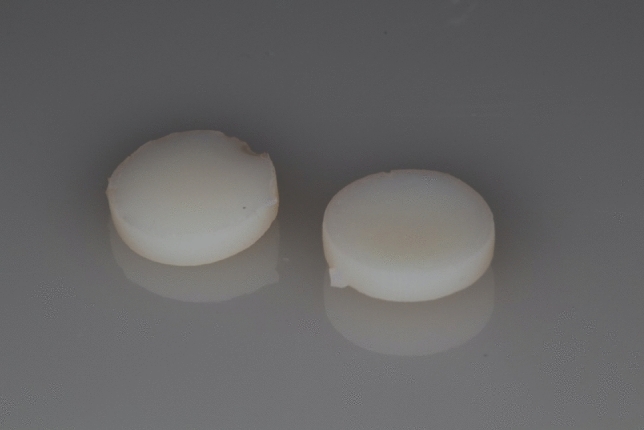
Representation of CB sample at baseline (left) and after 1 day aging (right)

Color stability results (Fig. 3) showed significant results for resin (*p* < 0.001) and time (*p* < 0.001). The interaction effects between resin and time (*p* = 0.029), and between resin and post-curing method (*p* < 0.001) were also found to be significant. No differences were observed for resin and manufacturing mode (*p* = 0.87), or the interaction effect between resin, manufacturing method and post-curing method (*p* = 0.35). In general, OnX showed a higher degree of color change than PC2, longer storage times resulted in increased color change for both materials, and CB post-cured with VG showed lower color alteration than CB cured with PC2. In addition, PC2 showed higher color change than VG for CB only. Nevertheless, after 1 day aging, there were no differences between manufacturing method considering same resin and curing methods. For CB, samples cured with VG showed lower color alteration, although the only differences were observed comparing not printed samples cured with PC2 and printed samples cured with BG. For OnX, all samples showed similar degree of color alteration. Figure 4 shows a representation of CB sample at baseline (left) and after 1-day aging (right).

## Discussion

This study focused on evaluating two completely distinct situations: samples 3D printed with a fully validated, closed workflow printer, following all recommended manufacturer instructions; or not printed at all. This workflow allowed evaluation of the capabilities of the post-curing protocols without any possible interference of the printing process, in order to determine if a 3D printer could influence the physical–mechanical properties of a 3D printed part if a capable post-curing unit were to be used.

Flexural strength results were higher for OnX when compared with CB resin (Table 2), regardless of the manufacturing method or post-curing protocol. Such results suggest the post-polymerization using VG for 120 s or PC2 following the recommended protocol is sufficient to achieve adequate polymerization, regardless of the printing parameters. While PC2 is part of a fully validated 3D printing workflow, post-polymerization using VG for 120 s has been reported to result in similar or enhanced properties when compared with other curing chambers, supporting the present results [6]. The higher results for OnX are compatible with the manufacturers’ provided information, with another study (131.0 ± 11.6 MPa) [23], and also correlate with the material’s indications. Although both materials are indicated for provisional restorations, OnX is also indicated for temporaries in hybrid/ “All-on-X” cases, which normally would require enhanced mechanical properties than regular resins. CB resin showed similar flexural strength when compared to other reports in the literature assessing different 3D printed temporary materials with results ranging between 90 and 130 MPa [6, 13, 22–24]. The range of results can be explained by the different used methodologies and materials. It is noteworthy one of the cited studies [6] compared different resins cured with Formcure curing unit (Formlabs), an experimental curing chamber, and Valo Grand for 40–120 s. Similarly to this study results, Valo Grand for 120 s resulted in similar or increased properties when compared with the other curing units.

Color stability results followed the trend observed for flexural strength (Fig. 2). In general, OnX showed a higher degree of color change than CB, longer storage times resulted in increased color change for both materials, and CB cured with VG showed lower color alteration than CB cured with PC2. Similar or better color stability after curing with VG for 120 s were similar to another study [6]. Despite the lack of studies reporting color stability for OnX and CB, degree of color change ranging from 4 to 9 after artificial aging is similar to other reports in the literature [6, 7, 12, 22]. Considering the fact that no staining solution was in contact with the evaluated resins, the color changes resulted from differences in the final polymer network structure.

It is interesting to observe similar color stability between printed and non-printed samples considering that non-printed samples were never washed with IPA to remove the excess of uncured material. Such observation could indicate that the 3D printed parts have inherently a lower color stability when compared with conventional resin composites [12], or that there is still significant amount of uncured monomer after the post-curing processes.

To the best of the authors’ knowledge, this is the first study comparing a fully validated 3D printing workflow with non-validated workflows. Despite a study reported similar or better results when using VG for 120 s compared to other curing chambers, some of the materials were not designed to be used with Formlabs 3D printers [6]. The present results demonstrated that VG provides similar or enhanced physical and mechanical properties when compared with PC2, which is a validated post-curing chamber. These results are in agreement with another study [6]. In addition, parts printed with the Pro95 3D printer were similar to non-printed samples.

It is noteworthy that only 2 resins and 1 curing chamber and light curing unit were evaluated. Although the present results considering the post-curing protocols appear to have a similar trend to previous studies, in which the post-polymerization process seems to be the most important step to achieve optimal properties, results may vary depending on the tested resin [2, 3, 6, 7, 9, 13, 14, 16–22]. In addition, there is a wide variety of curing chambers available in the market, meaning that the printing process could potentially influence 3D printed parts properties if a curing chamber with lower light intensity were to be used.

In addition, although the physical–mechanical properties seem to rely more on the post-curing process than on the 3D printer itself, it is necessary to better evaluate the effects of different 3D printers and parameters in the printing accuracy, which was not the objective of the present study. Therefore, future studies should evaluate different resins and curing chambers, as well as the effect of the printing and post-curing parameters in the final dimensions and biological properties of 3D printed parts.

Based on the limitations of the present study, the manufacturing method (3D printed or not 3D printed) does not seem to influence the flexural strength and color stability of 3D printed resins. Therefore, at least from a physical–mechanical perspective, the final properties of the material are dependent mainly on the post-polymerization process, provided an adequate post-curing protocol is used.

## Data Availability

Data from this study is available upon request.
